# Unmasking Sarcoidosis as the Culprit Behind Hypercalcemia-Induced Acute Pancreatitis: A Diagnostic Conundrum

**DOI:** 10.7759/cureus.47643

**Published:** 2023-10-25

**Authors:** Aditya Kohli, Anshdeep Saluja, Muzammil Farooqi, Saurabh Arora, Naveen Mittal

**Affiliations:** 1 Internal Medicine, Dayanand Medical College and Hospital, Ludhiana, IND; 2 Medicine, Dayanand Medical College and Hospital, Ludhiana, IND; 3 Endocrinology, Dayanand Medical College and Hospital, Ludhiana, IND; 4 Endocrinology and Diabetes, Dayanand Medical College and Hospital, Ludhiana, IND

**Keywords:** hilar lymphadenopathy, multiorgan sarcoidosis, extrapulmonary sarcoid, granulomatous disorder, multiple lung nodules, calcitonin, corticosteroids, acute pancreatitis, sarcoidosis hypercalcemia, pancreatitis due to hypercalcemia

## Abstract

Hypercalcemia-induced pancreatitis is a rare and challenging complication, particularly when secondary to sarcoidosis. This case report discusses the clinical presentation, diagnostic workup, and management of a 61-year-old patient diagnosed with hypercalcemia-induced pancreatitis secondary to sarcoidosis. The abstract highlights the complexities of diagnosing pancreatitis linked to elevated calcium levels and underscores the importance of recognizing underlying conditions such as sarcoidosis in these cases. Through this case, we aim to enhance awareness among clinicians regarding the association between sarcoidosis, hypercalcemia, and pancreatitis, ultimately contributing to improved diagnostic accuracy and patient care strategies.

## Introduction

Sarcoidosis, a multisystem inflammatory disorder characterized by non-caseating granulomas, can affect virtually any organ system within the body. While its etiology remains elusive, sarcoidosis often presents with a myriad of clinical manifestations, making it a diagnostic challenge for clinicians [1]. Hypercalcemia, a relatively common complication of sarcoidosis, results from the dysregulated activation of macrophages, primarily within the affected organs, leading to the excessive production of 1,25-dihydroxyvitamin D [2,3]. This, in turn, causes increased intestinal calcium absorption and bone resorption. The consequences of hypercalcemia in sarcoidosis can range from mild, nonspecific symptoms to severe, life-threatening complications. While alcohol and gallstones are the most common etiologies of acute pancreatitis, non-traditional causes, such as hypercalcemia secondary to sarcoidosis, can lead to acute pancreatitis [4,5]. This case report describes an intriguing clinical encounter with a patient who developed acute pancreatitis as a result of sarcoidosis-induced hypercalcemia [6]. We present the clinical presentation, diagnostic workup, and management of this unique case, shedding light on the complex interplay between sarcoidosis, hypercalcemia, and acute pancreatitis. Furthermore, this report emphasizes the importance of a multidisciplinary approach in the evaluation and treatment of sarcoidosis patients with unusual and potentially life-threatening complications.

## Case presentation

A 61-year-old male, non-alcoholic and non-smoker, presented to the OPD with excruciating upper abdominal pain and vomiting for two days. The pain was localized to the mid-epigastric region, stabbing in nature, and radiating to the back. The patient reported three episodes of non-bilious, non-projectile vomiting that was yellowish in color and mixed with food particles. The patient had episodes of dry cough for the past three weeks and complained of a low-grade fever with a loss of appetite. The patient had tachycardia and was in distress due to severe abdominal pain. Vitals were recorded with a temperature of 99.4 °F, a pulse rate of 106 beats per minute, a respiratory rate of 21 breaths per minute, and a blood pressure of 124/84 mmHg. On abdominal examination, there was tenderness to palpation in the epigastric region. Systemic examination and review of systems were negative, with non-contributory findings.

Investigations

**Figure 1:**
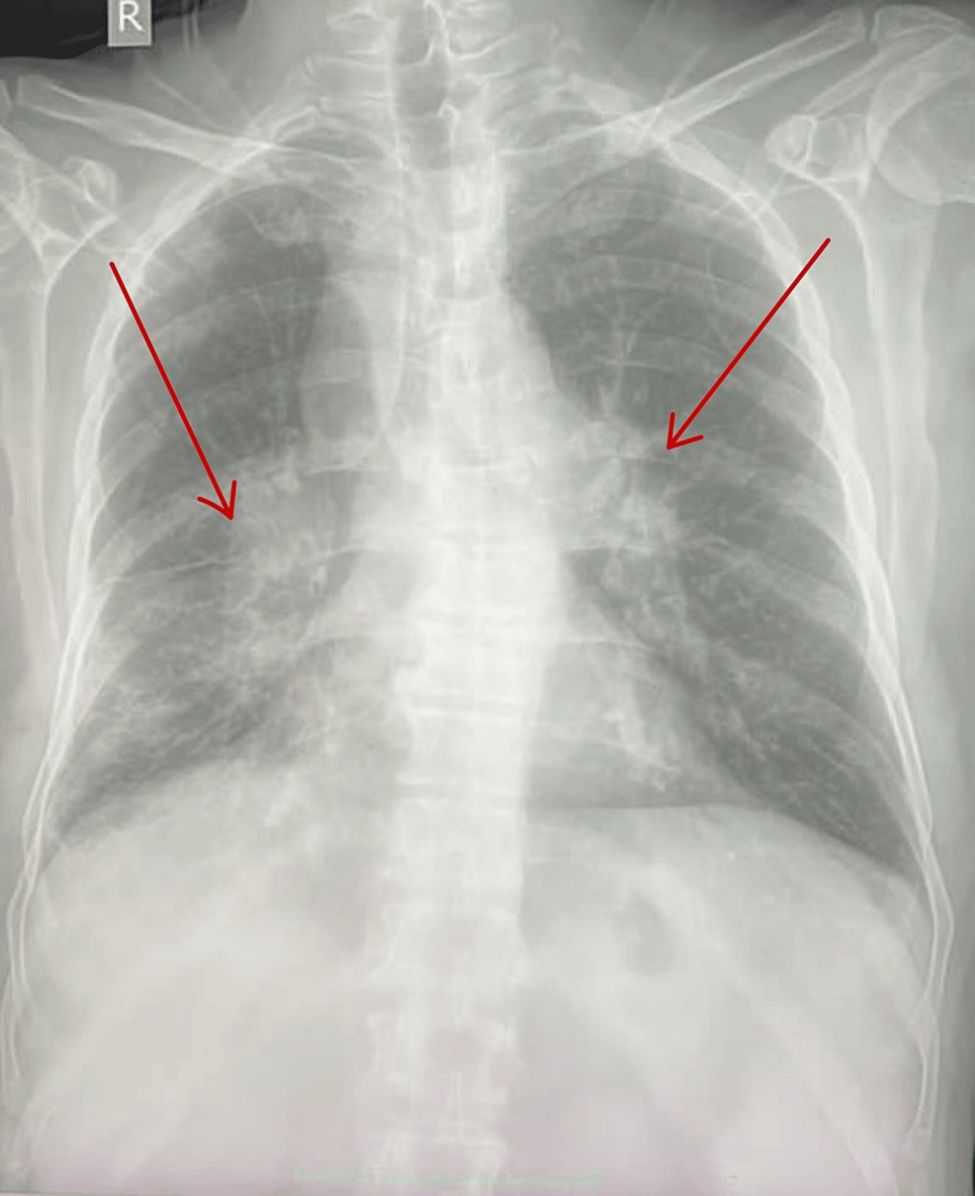
Chest X-ray showing hilar lymphadenopathy *Findings are marked with a red arrow.

**Figure 2:**
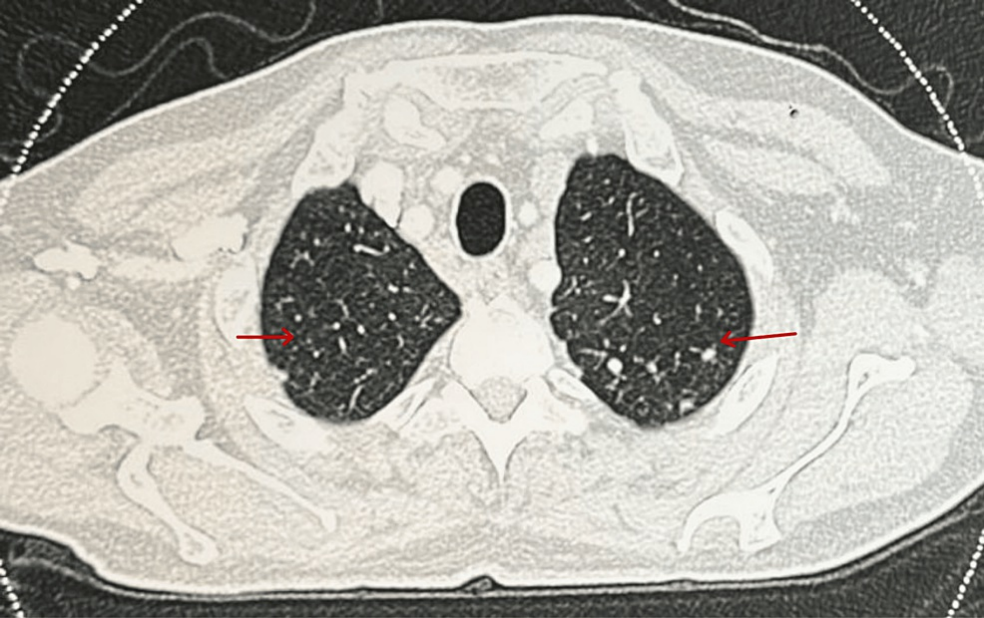
MDCT chest showing lung nodules and mediastinal lymphadenopathy *Findings are marked with a red arrow.

**Figure 3:**
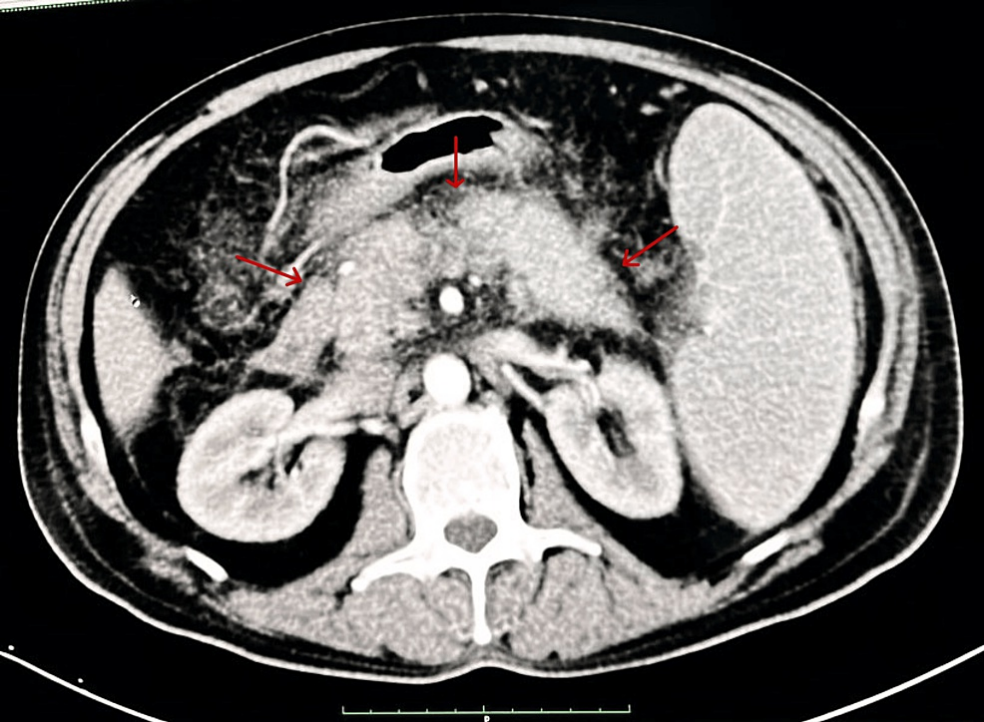
CECT abdomen showing a diffuse pancreatic enlargement with heterogenous enhancement and peripancreatic stranding *Findings are marked with a red arrow.

**Figure 4:**
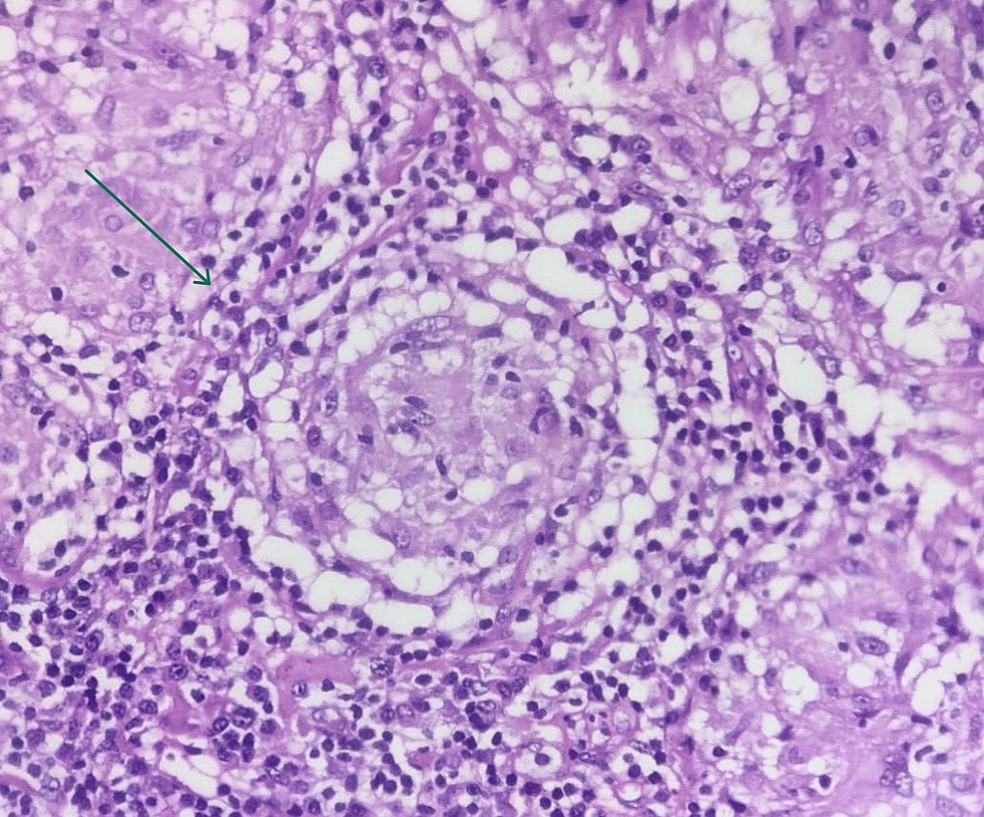
Histopathological examination showing non-caseating granulomas *Findings are marked with a green arrow

The initial evaluation revealed significantly elevated serum amylase and lipase levels. A comprehensive metabolic panel was shown to have elevated serum calcium levels and deranged renal function tests. Vitamin D3 levels were normal with suppressed parathormone levels (Table 1). USG abdomen revealed hypoechoic and bulky pancreas measuring 3.1 cm and 3.8 cm in the head and proximal body regions, respectively. A small amount of peripancreatic fluid with increased echogenicity of peripancreatic fat was seen along with grade-I fatty liver. Chest X-ray (CXR) showed hilar lymphadenopathy and bilateral reticular opacities (Figure 1). Multidetector computed tomography scan (MDCT) of the chest revealed multiple subpleural nodules with fissural nodularity along the right major fissure. The right lung showed diffuse peribronchial thickening with nodularity of the peribronchovascular and perilymphatic intersitium. Extensive large discrete and conglomerated enhancing lymph nodes with a central non-enhancing component were seen in prevascular, pretracheal, paratracheal, precarinal, subcarinal, and aortopulmonary windows and bilateral hilar locations measuring up to 37 mm in a short-axis diameter (Figure 2). Contrast-enhanced CT scan (CECT) of the abdomen revealed multiple enlarged lymph nodes at the porta hepatis and along the celiac axis. The pancreas was bulky in size, with heterogenous enhancement and extensive stranding in the peripancreatic region (Figure 3). Further workup included transbronchial lung biopsy and histopathological examination, which revealed non-caseating granulomatous lymphadenitis (Figure 4). A final diagnosis of hypercalcemia-induced acute pancreatitis was made, secondary to sarcoidosis.

**Table 1:** Laboratory evaluation (initial presentation, day five of admission, and on discharge) PTH: Parathormone; CRP (Q): C-reactive protein (quantitative); ACE: Angiotensin-converting enzyme; HDL-C: High-density lipoprotein cholesterol; LDL-C: Low-density lipoprotein cholesterol; N.A: Not available

Labs	On Admission	Day 5 post-calcitonin therapy	On discharge	Reference range
Total Leucocyte Count	8,300/mm^3^	6,300/mm^3^	5,000/mm^3^	4000-11,000/mm^3^
Haemoglobin	12.3 gm/dl	11.6 gm/dl	13.2 gm/dl	12-16 gm/dl
Platelet Count	1,05,000/mm^3^	1,39,000/mm^3^	2,12,000/mm^3^	1,50,000-4,50,000/mm^3^
RBC Count	3,59,000/mm^3^	3,12,000/mm^3^	3,80,000/mm^3^	4,50,000-5,50,000/mm^3^
PCV	36.5%	31.2%	37.1%	45.0-55.0 (%)
S. amylase	1493.0 U/L	956.0 U/L	230.0 U/L	28-100 U/L
S. lipase	1834.0 U/L	1278.0 U/L	178.0 U/L	13-60 U/L
S. creatinine	3.60 mg/dl	2.44 mg/dl	1.37 mg/dl	0.7-1.2 mg/dl
S. urea	78 mg/dl	56 mg/dl	22 mg/dl	10.0-50.0 mg/dl
S. calcium	14.7 mg/dl	9.6 mg/dl	10.5 mg/dl	8.6-10.2 mg/dl
S. phosphorus	5.4 mg/dl	4.8 mg/dl	4.3 mg/dl	2.7-4.5 mg/dl
PTH	4.75 pg/ml	5.93 pg/ml	11.76 pg/ml	15.0-65.0 pg/ml
Vitamin D3	15.23 ng/ml	N.A	14.28 ng/ml	20.0-40.0 ng/ml
CRP (Q)	66.56 mg/L	46.42 mg/L	18.67 mg/L	0.0-6.0 mg/L
ACE	73.61 U/L	N.A	77.82 U/L	65.0-115.0 U/L
Total Cholesterol	96.0 mg/dl	N.A	104.0 mg/dl	50.0-200.0 mg/dl
Triglycerides	165.0 mg/dl	N.A	172.0mg/dl	50.0–200.0 mg/dl
LDL-C	73.0 mg/dl	N.A	82.0 mg/dl	80.0–130.0 mg/dl
HDL-C	14.0 mg/dl	N.A	24.0 mg/dl	35.0-55.0 mg/dl

Differential diagnosis

The diagnosis of sarcoidosis often involves differentiating it from tuberculosis, a common alternative diagnosis. The Mantoux test yielded a negative result. A thorough patient history and examination, the absence of cavitary lesions on imaging studies, the presence of hypercalcemia, and the identification of non-caseating granulomas in histopathological findings aided in the diagnosis of sarcoidosis. There was no history of occupational exposure to silica, making a diagnosis of silicosis less likely despite the presence of lung nodules. The diagnosis of lymphoma and fungal infections such as histoplasmosis was ruled out by the absence of specific clinical features, constitutional B-symptoms, the presence of hypercalcemia, and diagnostic histology. Parathormone levels were suppressed, ruling out hyperparathyroidism as a cause of hypercalcemia. Given that the patient's lipid profile fell within the normal range, hypertriglyceridemia was eliminated as a potential underlying cause of acute pancreatitis. A comprehensive review of the patient's medication history was conducted, and none of the prescribed drugs were identified as possible triggers for acute pancreatitis. The presence of mediastinal lymphadenopathy, elevated calcium levels, and lung nodules pointed toward sarcoidosis. A final diagnosis of hypercalcemia-induced acute pancreatitis secondary to sarcoidosis was made.

Treatment and outcome

The patient was managed conservatively with intravenous fluids and kept at nil per oral. Subcutaneous calcitonin was administered, and the serum calcium levels normalized on day five of admission. Intravenous corticosteroids were initiated to treat sarcoidosis, the underlying cause of the patient’s presentation. The dose of steroids was gradually tapered, and the patient was shifted to oral steroids on discharge. The abdominal pain and episodes of vomiting subsequently resolved, and the patient was discharged in stable condition.

## Discussion

This case report highlights a rare but significant complication of sarcoidosis, namely, hypercalcemia-induced acute pancreatitis [7]. Sarcoidosis is a multisystem granulomatous disorder characterized by the formation of non-caseating granulomas in affected organs. While pulmonary involvement is the most common presentation, extrapulmonary manifestations can occur, involving virtually any organ system [8]. One of the less frequent but potentially severe complications of sarcoidosis is hypercalcemia, which results from increased production of 1,25-dihydroxyvitamin D by activated macrophages within granulomas [9]. Elevated serum calcium levels can lead to a range of clinical manifestations, including renal dysfunction, neurologic abnormalities, and, as demonstrated in this case, pancreatitis [10]. The pathophysiology of acute pancreatitis in the setting of hypercalcemia is thought to be multifactorial. Elevated calcium levels may lead to premature activation of pancreatic enzymes within the acinar cells, resulting in autodigestion and inflammation of the pancreatic tissue [11]. Additionally, hypercalcemia can impair microcirculatory flow in the pancreas, leading to ischemia and further tissue damage. Diagnosing sarcoidosis-induced hypercalcemia as the etiology of acute pancreatitis can be challenging, particularly in patients without a known history of sarcoidosis. A high index of suspicion is crucial, and it is recommended to perform a thorough evaluation for potential underlying causes of hypercalcemia, including measurement of parathyroid hormone (PTH) levels to differentiate between hyperparathyroidism and non-parathyroid mediated hypercalcemia [12,13]. In this case, the presence of mediastinal lymphadenopathy, coupled with the characteristic findings on chest CT, provided strong clinical evidence for the diagnosis of sarcoidosis [14]. Further confirmation was achieved through the identification of non-caseating granulomas on biopsy. Management of acute pancreatitis in the setting of sarcoidosis-induced hypercalcemia necessitates a multifaceted approach. Prompt rehydration and correction of electrolyte imbalances are paramount, along with aggressive pain control and the initiation of nutritional support [15]. Given the hypercalcemia, specific measures to lower serum calcium levels, including subcutaneous calcitonin, intravenous hydration, and corticosteroids, were instituted in this case. Long-term management of sarcoidosis-induced hypercalcemia involves addressing the underlying granulomatous process. Corticosteroids remain the mainstay of treatment, although their use should be carefully weighed against potential risks, especially in patients with comorbidities, such as diabetes mellitus [16]. Close monitoring of calcium levels, renal function, and bone health is imperative during treatment. This case report highlights the role of calcitonin and corticosteroids in the management of hypercalcemia-induced acute pancreatitis in a patient with concomitant sarcoidosis and acute kidney injury.

## Conclusions

In conclusion, this case highlights the importance of considering sarcoidosis-induced hypercalcemia as a potential cause of acute pancreatitis, especially in patients with characteristic clinical and radiologic findings suggestive of sarcoidosis. High clinical suspicion and appropriate diagnostic workup are essential for promptly diagnosing and managing such a unique case presentation. The complexities observed in this case underscore the need for further in-depth research into similar cases to unravel the underlying mechanisms and establish optimal diagnostic and therapeutic strategies. Additionally, exploring the long-term outcomes and quality of life in these patients post-treatment could enhance our understanding of the disease trajectory. Collaborative efforts between clinicians, researchers, and specialists are imperative to advance our knowledge in this area, potentially leading to the development of targeted therapies and improved patient outcomes.
